# Rice Dwarf Virus Small RNA Profiles in Rice and Leafhopper Reveal Distinct Patterns in Cross-Kingdom Hosts

**DOI:** 10.3390/v11090847

**Published:** 2019-09-12

**Authors:** Yu Wang, Rui Qiao, Chunhong Wei, Yi Li

**Affiliations:** State Key Laboratory of Protein and Plant Gene Research, College of Life Sciences, Peking University, Beijing 100871, China

**Keywords:** RNA silencing, vsiRNAs, rice dwarf phytoreovirus, RNA-dependent RNA polymerase

## Abstract

RNA silencing has evolved as a widespread antiviral strategy in many eukaryotic organisms. Antiviral RNA silencing is mediated by virus-derived small RNAs (vsiRNAs), created by the cleavage of double-stranded viral RNA substrates by Dicer (Dcr) in animals or Dicer-like (DCL) proteins in plants. However, little is known about how the RNA silencing mechanisms of different hosts respond to the same virus infection. We performed high-throughput small RNA sequencing in *Nephotettix cincticeps* and *Oryza sativa* infected with Rice dwarf phytoreovirus and analyzed the distinct accumulation of vsiRNAs in these two hosts. The results suggested a potential branch in the evolution of antiviral RNA silencing of insect and plant hosts. The rice vsiRNAs were predominantly 21 and 22 nucleotides (nt) long, suggesting that OsDCL4 and OsDCL2 are involved in their production, whereas 21-nt vsiRNAs dominated in leafhopper, suggesting the involvement of a Dcr-2 homolog. Furthermore, we identified ~50-fold more vsiRNAs in rice than in leafhoppers, which might be partially attributable to the activity of RNA-dependent RNA polymerase 6 (RDR6) in rice and the lack of RDR genes in leafhoppers. Our data established a basis for further comparative studies on the evolution of RNA silencing-based interactions between a virus and its hosts, across kingdoms.

## 1. Introduction

RNA silencing, or interference (RNAi), is a gene regulatory mechanism of eukaryotic organisms that acts as an innate antiviral immune response in invertebrates, plants, fungi, and mammals [1,2]. During antiviral RNAi, viral replicative double-stranded RNA (dsRNA) intermediates generated during viral RNA replication are recognized and cleaved by host Dicer enzymes into 21- to 24-nucleotide (nt) small interfering RNAs (siRNAs) [3]. Virus-derived small interfering RNAs (vsiRNAs) are then incorporated into Argonaute (AGO)-containing RNA-induced RNA silencing complexes (RISCs) to initiate the cleavage of cognate viral genes. Thus, viral infection induces the production of primary vsiRNAs. Biosynthesis and amplification of vsiRNAs are then dependent on the host or viral RNA-dependent RNA polymerases (RDRs) producing secondary vsiRNAs [3,4]. In the arms race between virus and host, many viruses have evolved viral suppressors of RNA silencing (VSRs) to counter the host antiviral RNAi pathway [5,6,7,8].

Many plant viruses are transmitted by insects and are thus capable of replicating in both plant and insect vector hosts. Persistent propagative plant viruses can be disastrous for agriculture [9,10] but confer only minor fitness costs to their insect vectors [11]. Antiviral RNAi in plants requires amplification of vsiRNA by host RDRs [12]. Rice (Oryza sativa) RDR6 (OsRDR6) plays an important role in the defense against Rice stripe virus (RSV) [13] and Rice dwarf phytoreovirus (RDV) [14]. Unlike plants, insects do not have endogenous RDRs, raising the question of whether there are differences between plant and insect RNAi antiviral pathways. While significant progress has been made in elucidating the molecular mechanisms of host silencing and viral anti-silencing in a number of virus–plant host systems [15], little is known about the RNAi antiviral pathways of insect vectors. More information is needed to achieve a more holistic understanding of virus transmission mechanisms and evolution of virus–host interactions, and to develop more effective antiviral strategies.

**Scheme 1:**
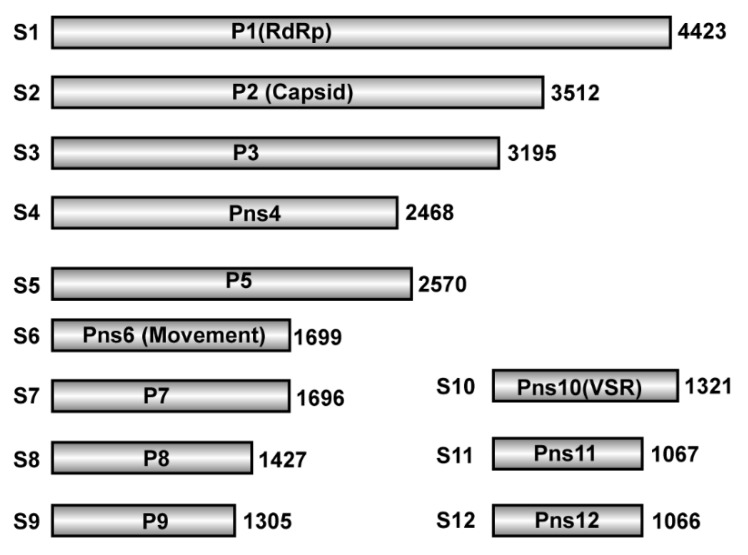
Genome structure of rice dwarf phytoreovirus (RDV).

RDV, a member of the genus phytoreovirus in the family *Reoviridae*, replicates *O. sativa* in its plant host. RDV is transmitted by the leafhopper insect vector *Nephotettix cincticeps* in a propagative manner. The RDV genome comprises 12 dsRNA segments (S1–S12) encoding seven structural proteins and five nonstructural proteins (Scheme 1) [16,17,18,19]. In this study, we used deep sequencing to characterize RDV-derived vsiRNAs in rice and leafhopper, two natural RDV hosts from different kingdoms, to develop a foundation for further studies on the evolution of RNA silencing-based relationships between a virus and its cross-kingdom hosts.

## 2. Materials and Methods

### 2.1. Rice Plant Growth, Insect Raising, and Cell Culture

*O. sativa* subsp. japonica ‘Zhonghua11′ was used as the wild type (WT) from which to generate OsRDR6 antisense transgenic plants (OsRDR6AS), as described previously [13]. Rice plants were grown in a greenhouse at 25 ± 1 °C under a 14:10 h (light:dark) photoperiod with a relative humidity of 60% to 70%. Green rice leafhoppers (*N. cincticeps*) were maintained in cages and reared on rice seedlings (Zhonghua11). Vector cells in monolayer (VCMs) derived from *N. cincticeps* were maintained in Kimura’s insect medium at 25 °C, as described previously [20].

### 2.2. Virus Inoculation

‘Zhonghua11′ rice plants were inoculated with RDV, as described previously [21]. In brief, 2-week-old rice seedlings were exposed to viruliferous leafhoppers for 48 h to acquire RDV. Plants were then removed from the leafhoppers and grown in a paddy field for analysis of RDV-induced symptoms and RNA extraction at 2 weeks post-inoculation (wpi) and 4 wpi. Adult leafhoppers were reared with RDV-infected rice plants for 2 days and then transferred to healthy rice seedlings. Leafhoppers were collected at 2 days post-inoculation (dpi), 6 dpi, and 10 dpi. The second instar larva leafhoppers were reared with RDV-infected rice for 2 days and then transferred to healthy rice. Then, the larva leafhoppers were collected at 3 dpi. VCMs were inoculated with purified RDV at a multiplicity of infection of 5 in a solution of 0.1 M histidine containing 0.01 M MgCl2 (pH 6.2; His-Mg) for 2 h at 25 °C. This solution was replaced with Kimura’s insect medium for culturing. At 2 days post inoculation, VCMs were collected using a trypsin-EDTA solution.

### 2.3. Small RNA Deep Sequencing

Total RNAs from each group were extracted using a Trizol reagent (Mf736-01; Mei5bio), according to the manufacturer’s instructions and were resolved on a 15% denaturing agarose gel. Gel slices containing fragments of 18 to 28 nt were excised, and the RNAs were eluted and purified for library construction (RK20403; ABclonal). Each library replicate contained RNA samples pooled from 200 leafhoppers, four plates of leafhopper VCMs, and 30 plants, respectively. Small RNA library preparation for sequencing using the Illumina platform was essentially performed as described previously [22]. 

### 2.4. Bioinformatics Analysis

A bioinformatics analysis was also performed as described previously [13]. Sequencing read adaptors were removed using the vectorstrip package. Small RNA reads with a length of 18 to 28 nt were mapped to the RDV genome using the BOAT software. Statistical analysis of small RNA data sets was performed using Perl scripts developed in-house. RNA-seq clean reads were mapped to the rice genome MSU7.0 using TopHat, and were analyzed using Cuffdiff. The Poisson-dispersion model of fragment was used to conduct statistical analysis ( false discovery rate< 0.05) and responsive genes were identified by reads per kilobase per million reads (RPKM). The hierachical clustering of the OsDCL and OsRDR genes was performed using the gplots package. 

### 2.5. RNA Northern Blotting

Northern blot analysis of virus-derived small RNAs and RDV genomic RNA was performed as described previously [9]. Probes partially complementary to the RDV genomic S11 and small RNA were labeled with α-32P-dCTP using a Random Primer DNA Labeling Kit (TaKaRa). Probe with a sequence complementary to U6 was used as a loading control. The Northern blot analysis probes of S11 were: S11-F1 (+): 5′-TCCGGGACCGGCTAACTCGACTGACCCACAGTGCCGATGCCTACCGACGACTGAATGACTTCGAAACAAGCATAATTTAG -3′; S11-R1(−):5′- AATGAGTGGAACATTACCCTTGGCTATGACGGCGAGTGAATCATTCGTTGGCATGCAAGTTTTGGCTCAAGACAAAGAAGTC -3′; vsiRNA (+): 5′-AGCCTTACTTACGCTTTGATT-3′; vsiRNA (−): 5′- GCTGCTTGATCACGTAGCTT-3′.

### 2.6. Accession Numbers

Small RNA data sets generated in this study have been deposited in the NCBI sequence read archive (http://www.ncbi.nlm.nih.gov/sra) under the accession number PRJNA541035. RNA-seq data of rice was obtained from Zhao et al. [10]. 

### 2.7. RNA Preparation and qPCR Analysis

Total RNA samples were prepared from rice and leafhoppers at differernt infectious stage, age, and tissues. Total RNA samples (2 µg) were treated with DnaseI (Promega, USA) to remove genomic DNA contamination and were used for cDNA synthesis (M-MLV Reverse Transcriptase Kit, Promega; Mir-X^TM^ miRNA First-Strand Synthesis Kit, Takara). The qPCR was performed using SYBR Green Real-Time PCR Master Mix (Mei5 Bioservices, China). *OsEF-1a* of rice and profilin of the leafhopper gene was used as an internal control for the assay, respectively. The Primers were: S2-F:5′-GCTATACACATCATCGCCGTGGTGT-3′; S2-R:5′-AACTTTGCTTCGGTGGTTGCCCCTG-3′; vsiRNA-F:5′ACATTTCTGACGTCGTTAGGA-3′; Profilin-F:5′GTGTTACCAAAGCCGCCATC-3′; Profilin-R5′TTGCCGAGTTTGGCTCTGAT-3′; OsEF1a-F 5′-ACATTGCCGTCAAGTTTGCTG-3′; OsEF1a-R 5′-AACAGCCACCGTTTGCCTC-3′.

## 3. Results

### 3.1. RDV Infection Triggers Abundant Production of vsiRNAs in Rice and Leafhopper

**Figure 1:**
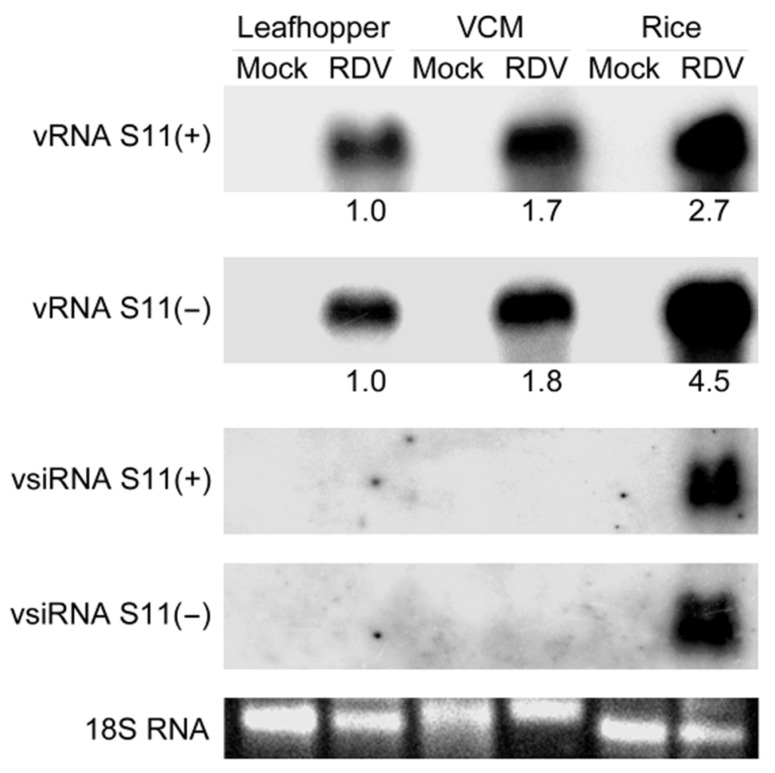
Accumulation of RDV genomic RNA S11 and vsiRNAs in different hosts. Northern blot assay of RDV genomic RNA and vsiRNAs in leafhoppers, vector cells in monolayer (VCMs), and rice. The 18S RNA served as a loading control. Mock—non-viruliferous insects or plants; RDV—viruliferous hosts; (+)—positive-strand RNA; (−)—negative-strand RNA; vRNA—RDV genomic RNA. Gel blot signals were quantified, and the relative intensities are shown below each lane.

The hallmark of antiviral RNAi response is the production of abundant vsiRNAs within infected cells [3]. Thus, we first investigated the production of vsiRNAs within RDV-infected plant and insect hosts. We infected adult leafhoppers, VCMs, and rice plants with RDV and subjected their total RNAs to Illumina-platform-based high-throughput sequencing of small RNA populations. We performed two biological replicates of each small RNA sequencing experiment and conducted further bioinformatics analysis, as described previously [13]. As shown in Table 1, the abundance of RDV vsiRNA was below 0.63% of the total small RNAs in leafhoppers and VCMs but were as high as 23.49% in rice plants. Thus, RDV vsiRNA accumulation was nearly 40- to 50-fold higher in rice than in leafhoppers and VCMs, respectively. To further examine this, we performed northern blot assays and found that the RDV vsiRNA band was strong in rice samples but invisible in leafhoppers and VCMs (Figure 1), consistent with the deep sequencing results. These results demonstrated that a significant amount of vsiRNA was produced in RDV-infected rice hosts but not in leafhopper hosts.

### 3.2. vsiRNAs Are Differentially Produced in Leafhopper and Rice Response to RDV Infection

Ruiz-Ruiz et al. [23] showed that a ~10-fold increase in accumulation of vsiRNAs derived from the citrus tristeza virus was positively correlated with a ~100-fold increase in the abundance of viral genomic RNAs in different plant hosts. Thus, we wondered whether the 40- to 50-fold greater accumulation of RDV vsiRNA in rice compared to leafhoppers and VCMs was correlated with different abundances of RDV genomic RNAs in rice and the insect hosts. However, semi-quantification of blot signals using ImageJ (version 1.4) revealed that the RDV genomic RNAs accumulated to similar levels in rice, leafhoppers, and VCMs (Figure 1). Furthermore, we performed qRT-PCR to examine the difference in relative RDV genomic RNAs and vsiRNA titer between rice and leafhopper, at different infectious stage, age, and tissues (Figure S1). The results from these analysis were consistent with the above conclusion. Thus, the distinct accumulation of RDV vsiRNAs in rice but not in insects was more likely due to differences between the RNA silencing machineries in these two hosts, rather than differences in the viral genomic RNA level. 

### 3.3. Distribution of vsiRNAs on RDV Genomic RNAs from RDV-Infected Leafhopper and Rice Hosts

**Figure 2:**
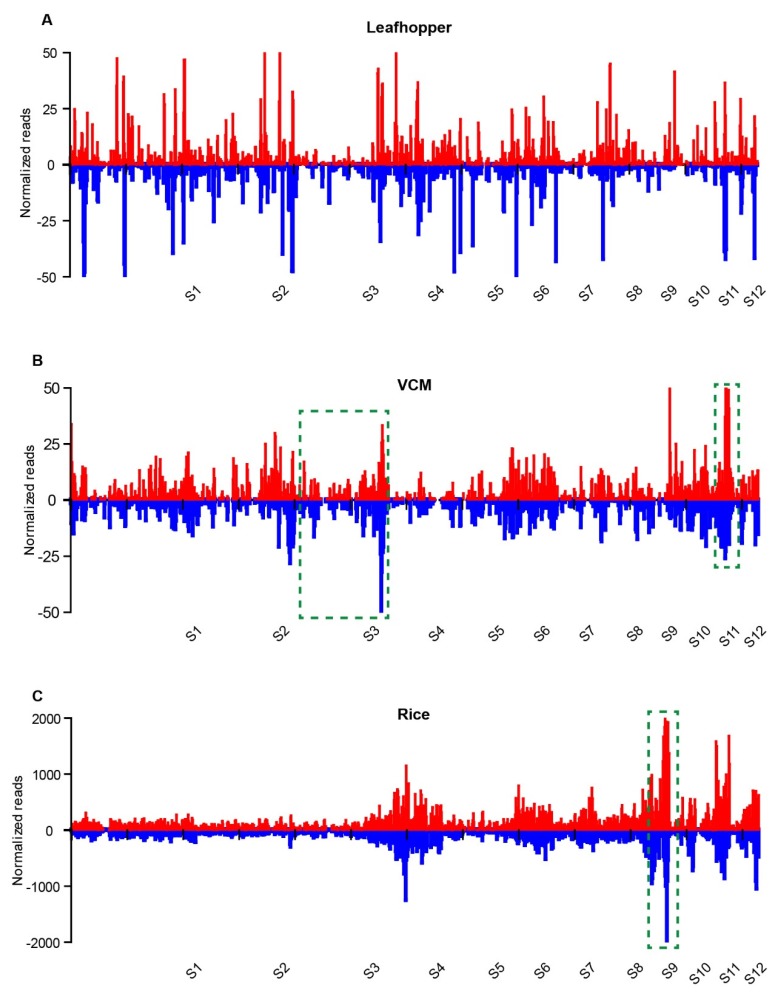
Genomic view of RDV vsiRNAs from RDV-infected leafhoppers (**A**), VCMs (**B**), and rice plants (**C**). vsiRNAs per million total sequenced small RNAs are plotted against the positive (**red**) or negative (**blue**) strands of the 12 RDV genomic RNAs (S1–S12) using a 20-nt window. Green boxes on S3 and S11 in VCM and S9 in rice represent the hotspots of vsiRNAs clustered at the terminal regions of genomic RNAs.

We further analyzed the distribution of vsiRNAs on various RDV genomic RNAs from RDV-infected leafhoppers and rice. S1 in leafhopper, S11 in VCMs, and S9 in rice produced the most abundant vsiRNAs (Figure 2 and Figure S2). The mechanisms underlying these differences remain to be explored. We also found that positive- and negative-strand vsiRNAs occurred in a nearly 1:1 ratio for each of the 12 genomic RNAs in leafhoppers, VCMs, and rice (Figure 2). This equal accumulation of positive- and negative-strand RDV vsiRNAs in both plant and insect hosts is similar to that of the flock house virus (FHV) vsiRNAs in Drosophila melanogaster [24]. Thus, RDV vsiRNAs most likely originated from dsRNA substrates, such as viral replicative intermediate dsRNAs or viral genomic dsRNAs. Several vsiRNAs hot spots were detected on different RDV genomic RNAs in both plant and insect hosts (Figure 2). Several of these vsiRNAs hot spots clustered at the terminal regions of genomic RNAs, including S3 and S11 in VCMs and S9 in rice (Figure 2), suggesting that replicative intermediate dsRNAs might be one of the main sources for RDV vsiRNA biogenesis.

### 3.4. Characterization of Virus-Derived Small RNAs in Cross-Kingdom Hosts Infected with RDV

**Figure 3:**
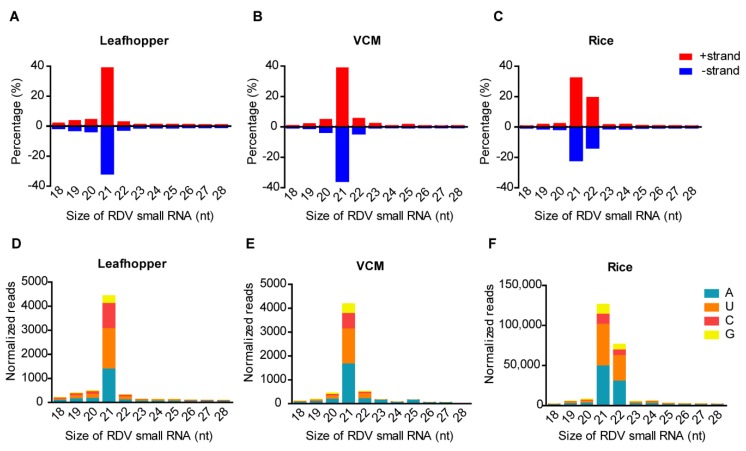
vsiRNA profiles of RDV-infected leafhoppers, VCMs, and rice plants. (**A**–**C**) Size distributions of RDV-derived small RNAs in the libraries from each species based on deep sequencing data. Percentages of positive-strand (top red bars) and negative-strand (bottom blue bars) vsiRNAs in the length range of 18 to 28 nt are shown. (**D**–**F**) Base preference for 5′-terminal first nucleotide of vsiRNAs of each length in the three libraries. A, U, C, and G represent adenine, uracil, cytosine, and guanine, respectively.

Since certain features of vsiRNAs reflect the potential molecular machinery for vsiRNA biogenesis in different hosts, we performed a bioinformatics analysis to characterize the vsiRNAs of RDV in both plant and insect hosts, in detail. vsiRNAs mapping to both the positive and negative strands of RDV genomic RNAs were predominantly 21 nt long in leafhoppers and VCMs (Figure 3A,B) but a mixture of 21 and 22 nt in rice (Figure 3C). Studies in flies and mosquitoes have shown that vsiRNAs derived from positive-strand RNA viruses, such as FHV, the Sindbis virus (SINV), and the Dengue virus, are usually 21 nt in length, and are produced by Drosophila endonuclease Dcr-2 and its orthologous protein in mosquitoes [24,25,26]. The presence of Dicer-2 and its homologs in leafhoppers have been verified by RNAi knockdown [27], and they might be involved in the production of RDV vsiRNAs. In Arabidopsis thaliana, AtDCL4 and AtDCL2 are responsible for producing 21- and 22-nt vsiRNAs, respectively, in a hierarchical manner [28]. Their homologs in rice, *OsDCL4* and *OsDCL2* [29,30], are induced by RDV infection (Figure S3). This result indicates that OsDCL4 and OsDCL2 might similarly be involved in the production of RDV vsiRNAs. Our further analysis revealed a strong bias in both hosts for sequences of RDV vsiRNAs with a 5′-terminal adenosine (A) or uridine (U) (Figure 3D–F). In Drosophila and Caenorhabditis elegans [31,32], small RNAs with a 5′ A substitution have been found to be attached to AGO2 protein [33]. vsiRNAs are loaded into the RISC containing AGO2 to specifically cleave viral mRNA, which is a critical step for the antiviral immune response mediated by RNA silencing [3]. Thus, AGO2 might also be involved in the loading of RDV vsiRNAs to form the functional antiviral RISC in both rice and leafhopper.

### 3.5. OsRDR6 Is Involved in RDV-Derived Small RNAs Production in Rice Plants

**Figure 4:**
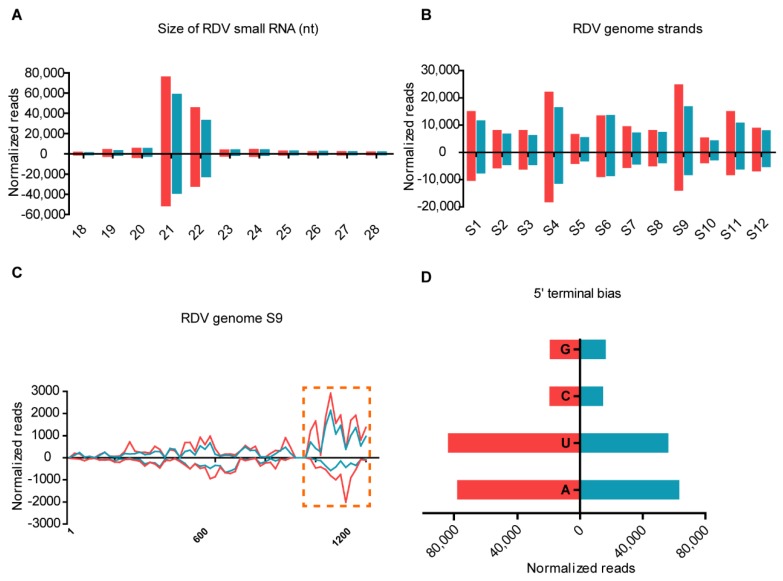
Comparison of vsiRNAs in response to RDV infection between OsRDR6AS and wild type (WT) plants. (**A**) Size distribution of RDV vsiRNAs. Normalized reads of 18- to 28-nt RDV vsiRNAs are shown. (**B**) Abundance of RDV vsiRNAs mapped to each of the 12 RDV genomic RNAs. (**C**) Location of vsiRNAs from the two libraries in RDV genomic RNA S9. (**D**) 5′-terminal first nucleotide bias in the two libraries. Red bars or lines indicate WT; blue bars or lines indicate OsRDR6AS.

Like its homologs in plants such as Arabidopsis [34,35] and Nicotiana benthamiana [36,37], OsRDR6 is involved in the biosynthesis of vsiRNAs from RSV in rice [13]. Here, we tested whether OsRDR6 is involved in the production of RDV vsiRNAs. We inoculated OsRDR6 knockdown (OsRDR6AS) rice plants with RDV. Small RNA deep sequencing data revealed reduced accumulation of RDV vsiRNAs in OsRDR6AS, compared to the WT plants (Table 1). We found that the expression of *OsRDR6* was reduced in RDV infected WT rice as compared to the mock control through RNA-seq analysis (Figure S3). Hong et al. [14] showed that the accumulation level of the OsRDR6 protein became undetectable after an RDV infection. Further data analyses showed that the length and polarity profiles of these vsiRNAs in virus-infected OsRDR6AS were less abundant than WT plants (Table 1 and Figure 4A). Meanwhile, analysis of the genomic distributions of RDV vsiRNAs showed that the vsiRNAs derived from S4 and S9 were clearly less abundant in infected OsRDR6AS than in WT plants, whereas those from S6 were not (Figure 4B). Detailed analysis indicated that vsiRNAs from the S9 3′-terminal region displayed the most dramatic reduction in OsRDR6AS as compared to the WT plants (Figure 4C). vsiRNAs from the S10, S11, and S12 3′-terminal regions also displayed clear reductions in OsRDR6AS plants, compared to the WT (Figure S4). Analysis of 5′-terminal nucleotide bias showed that vsiRNAs mostly begin with an A or U (Figure 4D). These data suggest that OsRDR6 plays an important role in the production of RDV vsiRNAs for the antiviral immune response in rice. Host RDR activities have not been identified in insect species; hence, OsRDR6 activity might contribute to the significantly higher accumulation of RDV vsiRNAs in rice than in leafhoppers and VCMs (Table 1).

More specifically, the intraparticle synthesis of reoviruses in the viroplasm suggests that their genomic dsRNAs are protected from antiviral dsRNA sensors [3,38]. Thus, RDV genomic dsRNAs and replicative intermediates might not be the major substrates for vsiRNA production. In rice, amplification of viral RNAs by OsRDR6 in the cytoplasm might produce many dsRNA substrates that are directly cleaved by the DCL2 protein to produce vsiRNAs. The lack of host RDR and viral RDR protein activities in the cytoplasm of insect cells could be one explanation for the low accumulation of RDV vsiRNAs in insect hosts as compared to plant hosts.

## 4. Discussion

Biological interactions between viruses and hosts involve many pathways. The RNAi pathway is a broad antiviral strategy present in cross-kingdom hosts [1]. Numerous studies have documented Dicer-dependent production of vsiRNAs in plants and insects after infection with a wide range of RNA viruses [1,39]. However, how different patterns of virus infection induce RNAi-mediated antiviral immunity in cross-kingdom hosts is unclear. Here, we used the phytoreovirus RDV and its different hosts (rice and leafhopper) as a model to better understand the underlying mechanisms. 

We used high-throughput small RNA sequencing combined with northern blotting to analyze vsiRNAs in rice and leafhoppers in response to RDV infection. Strikingly, RDV infection triggers abundant production of vsiRNAs in both rice and leafhopper hosts. We compared the characteristics of vsiRNAs between rice and leafhoppers, and observed (1) 50-fold higher levels of RDV vsiRNA in rice than in leafhoppers and VCMs; (2) a substantially different distribution of vsiRNA in RDV genomic RNAs in insect and plant hosts—more vsiRNAs produced from S1 in leafhopper, S11 in VCMs, and S9 in rice than from other genomic segments, with an almost equal accumulation of positive- and negative-strand RDV genomic RNAs; and (3) vsiRNAs of predominantly 21 nt in leafhoppers and VCMs, and 21 and 22 nt in rice. The positive- and negative-strand RDV vsiRNAs were mapped at a nearly 1:1 ratio to RDV genomic RNAs in leafhopper, VCMs, and rice. Therefore, we propose that the dramatically different characteristics of RDV vsiRNAs in rice and insects are more likely due to differences in molecular interactions between the RNA silencing machinery and the viral RNAs in these hosts than due to differences in viral genomic RNA levels. 

In plants, vsiRNAs are amplified by a family of related host RNA-dependent RNA polymerases known as RDRs [12]. The production of primary vsiRNAs processed from viral dsRNA replicative intermediates is necessary to trigger the RDR-dependent biogenesis of secondary vsiRNAs. No insect genes encode RDR homologs [40], possibly indicating why vsiRNA levels were much lower in leafhoppers than in rice, in response to an RDV infection. Our results showed reduced accumulation of RDV vsiRNAs in the infected OsRDR6AS rice compared to the WT, but still more than that in leafhopper. A previous study found that vsiRNAs accumulate to similar levels in the WT plants and rdr1, rdr2, and rdr6 triple-knockout mutant plants [41]. Additionally, RDR6-deficient plants are more susceptible to some RNA viruses than WT plants [42,43,44]. RDR6 plays a vital role in vsiRNA amplification for antiviral silencing [12,13,45,46]. Thus, we propose that vsiRNA amplification in rice might occur through homologous RDRs.

Antiviral RNAi defense against FHV in Drosophila involves 21-nt vsiRNAs produced by Dicer-2. However, in Arabidopsis, DCL4 and DCL2 produce 21- and 22-nt vsiRNAs, respectively [29]. A Dcr-2 homolog has been identified in leafhoppers and might be involved in the production of RDV vsiRNA [28]. Orthologs of DCL4 and DCL2 in rice have also been identified [47,48] and might be similarly involved in the production of RDV vsiRNAs. Furthermore, the individual knock-down of several rice *DCL*s (*DCL1*, *2*, *3a*, *3b* and *4*) are more susceptible to RSV infection than the WT plants, indicating a critical role for DCLs in rice antiviral defense [49]. Therefore, Dicer enzymes have a vital function in antiviral RNAi as specific vsiRNAs producers.

Another possible, and not necessarily mutually exclusive, explanation for the low accumulation of RDV vsiRNAs in leafhoppers and VCMs is the action of RDV VSR Pns10. Co-expression of FHV-encoded VSR B2 in SINV-injected mosquitoes causes a great reduction in the abundance of vsiRNAs [25]. Pns10 is indispensable for RDV replication in insect cells; it continued to be produced in VCMs during 6 years of maintenance after initial inoculation, in contrast to its disappearance from infected rice plants during the same period [50]. These possible causes of low vsiRNA levels in insects will be investigated in future studies. 

In summary, our work provides the first insight, to our knowledge, into vsiRNA profiles in an insect vector infected by a plant virus. Comparison of vsiRNA profiles in natural plant and insect hosts of the virus showed major quantitative and size differences of these vsiRNAs. Such differences suggest that there are distinct molecular pathways for the biogenesis of vsiRNAs from the same virus in hosts belonging to different kingdoms. Our findings establish a basis for further cross-kingdom comparative studies on the evolution of RNA silencing-based molecular interactions between a virus and its hosts.

## Figures and Tables

**Table 1:** Summary of deep sequencing results of small RNAs from RDV-infected leafhoppers, VCMs, and rice plants.

		Unique Sequences ^a^	Total Reads ^a^	Unique RDV vsiRNA ^a, b^	Total RDV vsiRNA Reads ^d^ (% of Total Reads) ^a, b^	(+)-Strands (%) ^a, c^	(−)-Strands (%) ^a, d^
Leafhopper	Replicate 1	1,322,051	6,998,353	12,212	44,014 (0.63%)	56.60%	43.40%
	Replicate 2	1,585,173	7,514,114	15,017	47,476 (0.63%)	56.73%	43.27%
VCM	Replicate 1	1,360,138	8,766,394	9,292	35,897 (0.41%)	56.34%	43.66%
	Replicate 2	1,219,667	7,837,090	8,394	36,411 (0.46%)	58.83%	41.17%
Rice	Replicate 1	2,610,966	6,999,547	144,347	1,637,665 (23.40%)	60.49%	39.51%
	Replicate 2	3,143,474	9,214,996	164,829	2,164,499 (23.49%)	61.14%	38.86%
OsRDR6AS	Replicate 1	1,996,171	6,283,390	112,334	1,112,734 (17.71%)	62.69%	37.31%
	Replicate 2	1,924,762	5,408,334	100,218	1,069,626 (19.78%)	62.90%	37.10%

^a^ Number of sequences within the set (18 nt ≤ length ≤ 28 nt).^b^ Sequences with perfect matches to the RDV genome. ^c^ Sequences with perfect matches to (+)-strand RNAs of the RDV genome. ^d^ Sequences with perfect matches to (−)-strand RNAs of the RDV genome.

## Supplementary Materials

The following are available online at https://www.mdpi.com/1999-4915/11/9/847/s1, Figure S1: The expression pattern of RDV S2 and vsiRNA in rice and leafhoppers infected with RDV, Figure S2: Total vsiRNA reads mapped to each of the RDV genomic RNAs, Figure S3: Expression profiles of OsDCL and OsRDR genes in rice infected with RDV, Figure S4: Distribution of vsiRNAs from RDV-infected OsRDR6AS and the wild type.
