# Supplementary material for: Rice Dwarf Virus Small RNA Profiles in Rice and Leafhopper Reveal Distinct Patterns in Cross-Kingdom Hosts
Source: Viruses. 2019 Sep 12;11(9):847. doi: 10.3390/v11090847 (PMC6784124; doi:10.3390/v11090847)
Supplement: Supplementary file 1 [file viruses-11-00847-s001.pdf]

Supplementary Figures

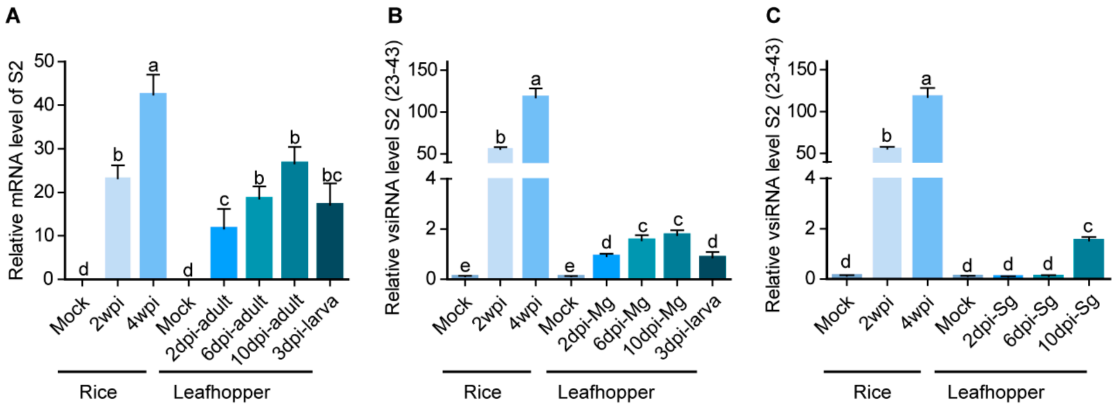

Figure S1. The expression pattern of RDV S2 and S2 derived vsiRNA in rice and leafhoppers infected with RDV. (A) mRNA level of RDV S2 in rice compared with leafhoppers were determined by qPCR. Rice were infected with RDV then examined at 2 weeks-post-inoculation (wpi) and 4 wpi. Leafhopper adults were infected with RDV then examined at 2 days-post-inoculation (dpi), 6 dpi and 10 dpi, respectively. Leafhopper larvas were infected with RDV then examined at 3 dpi. (B-C) vsiRNA level in rice compared with leafhoppers were determined by qPCR. The mid-gut (Mg) of leafhopper adults, (B) leafhopper larvas (B), and salivary gland (Sg) of leafhopper adults (C) were determined at 2 dpi to 10 dpi. Rice were determined at 2wpi and 4wpi. Tukey's honestly significant difference post hoc tests were performed for multiple comparisons. Letters indicate significant differences, $p<0.05$. Data are from three replicates.

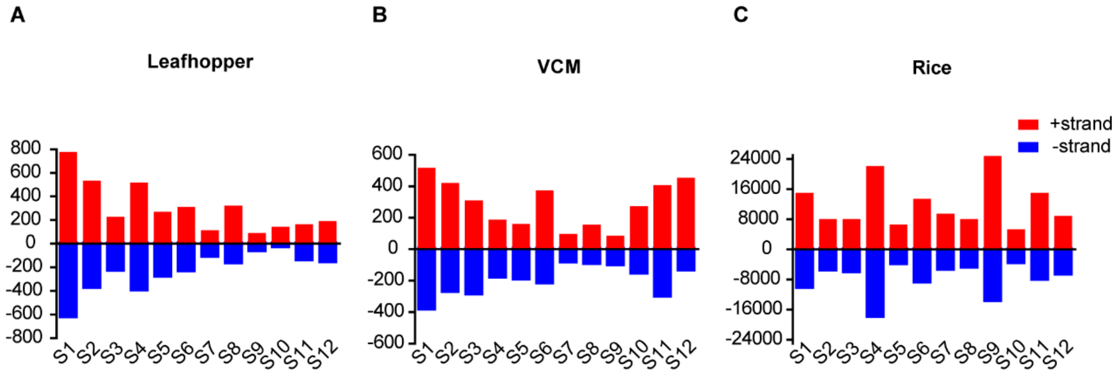

Figure S2. Total vsiRNA reads mapped to each of the RDV genomic RNAs. (A–C) Reads distribution of vsiRNAs in the 12 (S1–S12) viral genomic RNAs in RDV-infected leafhoppers (A), VCMs (B), and rice plants (C). Red and blue bars indicate positive-strand (top red bar) and negative-strand (bottom blue bar) RDV genomic RNAs.

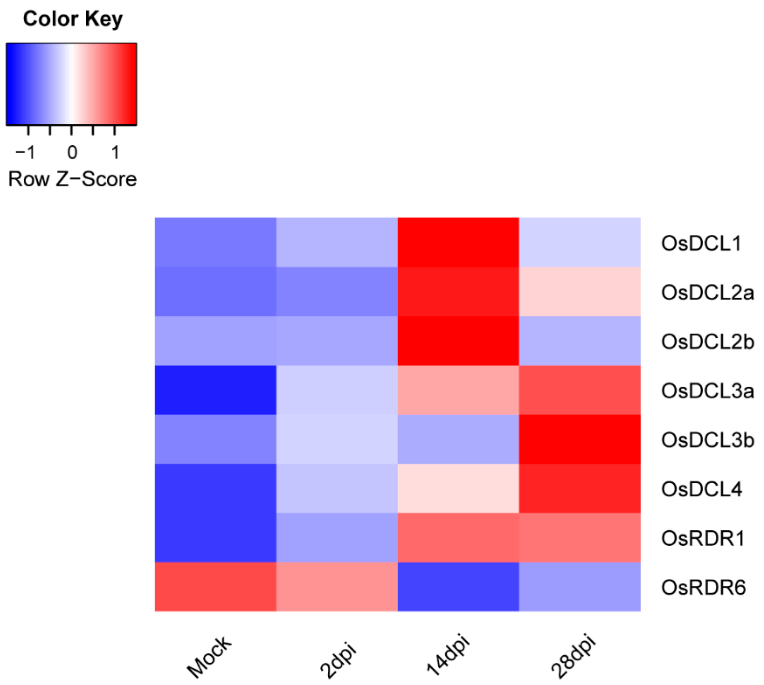

Figure S3. Expression profiles of OsDCL and OsRDR genes in rice infected with RDV. Rice was infected with RDV then examined at 2 dpi, 14dpi and 28dpi, respectively.

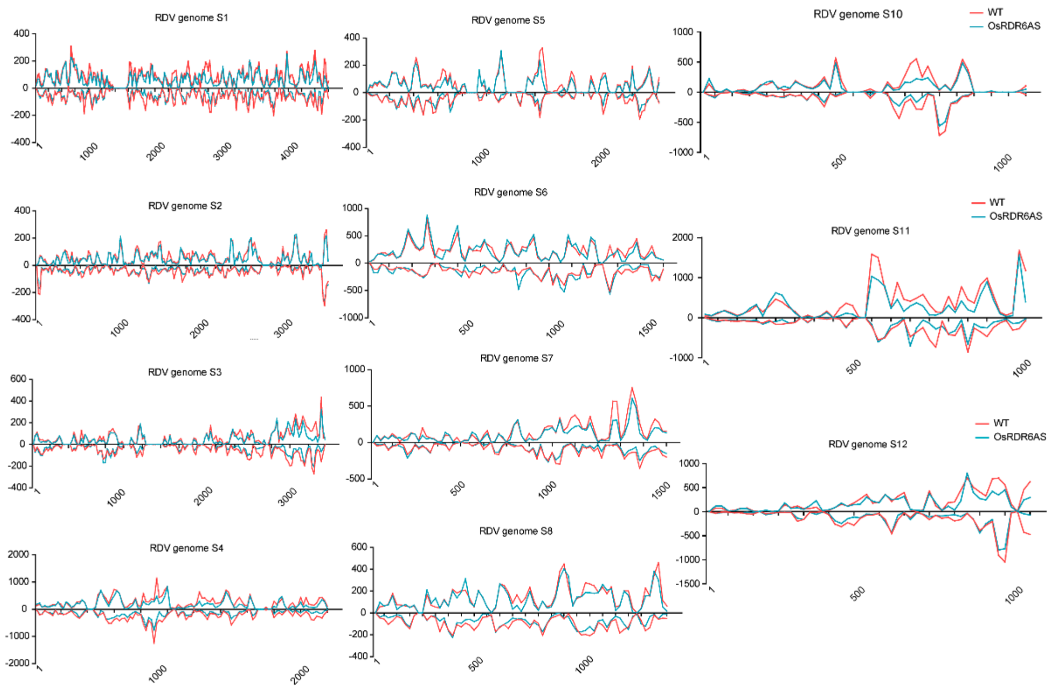

Figure S4. Distribution of vsiRNAs from RDV-infected OsRDR6AS and the wild type rice. vsiRNAs per million total sequenced small RNAs plotted against the RDV genomic positive-strand (top) or negative-strand (bottom) S1–8 and S10–12, respectively. Red lines indicate WT; blue lines indicate OsRDR6AS.
